# A phase II trial of 1st-line modified-FOLFOXIRI plus bevacizumab treatment for metastatic colorectal cancer harboring RAS mutation: JACCRO CC-11

**DOI:** 10.18632/oncotarget.24702

**Published:** 2018-04-10

**Authors:** Hironaga Satake, Yu Sunakawa, Yuji Miyamoto, Masato Nakamura, Hiroshi Nakayama, Manabu Shiozawa, Akitaka Makiyama, Kazuma Kobayashi, Yutaro Kubota, Misuzu Mori, Masahito Kotaka, Akinori Takagane, Masahiro Gotoh, Masahiro Takeuchi, Masashi Fujii, Wararu Ichikawa, Takashi Sekikawa

**Affiliations:** ^1^ Department of Medical Oncology, Kobe City Medical Center General Hospital, Kobe, Hyogo, 650-0047, Japan; ^2^ Department of Clinical Oncology, St. Marianna University School of Medicine, Kawasaki, Kanagawa, 216-8511, Japan; ^3^ Department of Gastroenterological Surgery, Graduate School of Medical Sciences, Kumamoto University, Kumamoto, 860-8556, Japan; ^4^ Aizawa Comprehensive Cancer Center, Aizawa Hospital, Matsumoto, Nagano, 390-8510, Japan; ^5^ Department of Surgery, Nagoya Medical Center, Nagoya, Aichi, 460-0001, Japan; ^6^ Department of Gastrointestinal Surgery, Kanagawa Cancer Center, Yokohama, Kanagawa, 241-0815, Japan; ^7^ Department of Hematology/Oncology, Japan Community Healthcare Organization Kyushu Hospital, Kitakyusyu, Fukuoka, 806-8501, Japan; ^8^ Department of Surgery, Nagasaki University Graduate School of Biomedical Sciences, Nagasaki, 852-8501, Japan; ^9^ Division of Medical Oncology, Department of Medicine, Showa University School of Medicine, Shinagawa-Ku, Tokyo, 142-8666, Japan; ^10^ Division of Clinical Oncology, Jichi Medical University, Shimotsuke-Shi, Tochigi, 329-0498, Japan; ^11^ Gastrointestinal Cancer Center, Sano Hospital, Kobe, Hyogo, 655-0031, Japan; ^12^ Department of Surgery, Hakodate Goryoukaku Hospital, Hakodate, Hokkaido, 040-8611, Japan; ^13^ Cancer Chemotherapy Center, Osaka Medical College Hospital, Takatsuki, Osaka, 569-8686, Japan; ^14^ Department of Clinical Medicine (Biostatistics), Kitasato University School of Pharmacy, Minato-ku, Tokyo, 108-8641, Japan; ^15^ Department of Digestive Surgery, Nihon University School of Medicine, Itabashi-ku, Tokyo, 173-8610, Japan; ^16^ Division of Medical Oncology, Showa University Fujigaoka Hospital, Yokohama, Kanagawa, 227-8501, Japan

**Keywords:** FOLFOXIRI, bevacizumab, colorectal cancer, RAS mutant, sidedness

## Abstract

FOLFOXIRI plus bevacizumab is considered a standard initial therapy for metastatic colorectal cancer (mCRC). However, few prospective trials have evaluated triplet therapy plus bevacizumab in patients with RAS mutant mCRC. Patients with an age of 20 to 75 years, and unresectable, measurable tumors harboring RAS mutation were given first-line treatment with bevacizumab (5 mg/kg on day 1) plus modified-FOLFOXIRI (irinotecan 150 mg/m^2^, oxaliplatin 85 mg/m^2^, levofolinate 200 mg/m^2^, and fluorouracil 2400 mg/m^2^ as a 46-h continuous infusion on day 1, repeated every 2 weeks). The primary endpoint was the objective response rate (ORR) as evaluated by an external review board. Progression-free survival (PFS), overall survival, early tumor shrinkage (ETS), depth of response (DpR), and safety were secondary endpoints. Among 64 patients who were enrolled between October 2014 and August 2016, 62 were evaluable for efficacy (right-sided tumors in 27%). ORR and disease control rate were 75.8% (95% confidence interval [CI] 65.1-86.5) and 96.8%, respectively. ETS was 73.8%, and median DpR was 49.2%. Median PFS was 11.5 (95% CI 9.5-14.0) months as of the cut-off date of September 2017. Adverse events of grade 3 or 4 were neutropenia (54%), hypertension (32%), diarrhea (13%), anorexia (11%), peripheral neuropathy (2%), and febrile neutropenia (5%). In conclusion, this prospective trial demonstrated for the first time that FOLFOXIRI plus bevacizumab is an active first-line treatment for patients with RAS mutant mCRC. Modified-FOLFOXIRI plus bevacizumab might become an alternative regimen of triplet chemotherapy for mCRC in Japan.

## INTRODUCTION

Randomized studies in patients with metastatic colorectal cancer (mCRC), including the TRIBE, STEAM, and CHARTA trials, have demonstrated that bevacizumab combined with infusional fluorouracil/levofolinate/irinotecan/oxaliplatin (FOLFOXIRI) is more effective than doublet plus bevacizumab treatment and is tolerated as first-line treatment [1–3]. The phase II OPAL trial showed that FOLFOXIRI plus bevacizumab was highly effective in terms of response rate, survival, and secondary resection rate in patients with molecularly unselected mCRC [4]. The randomized phase II STEAM trial of sequential or concurrent FOLFOXIRI plus bevacizumab versus FOLFOX plus bevacizumab in the United States showed comparable results to the TRIBE trial [2]. The CHARTA trial, which was conducted parallel to the TRIBE trial to compare the same 4-drug-protocol with FOLFOX plus bevacizumab as a control arm, supported the superiority of the triplet regimen [3]. The European Society for Medical Oncology (ESMO) consensus guidelines strongly recommended the triplet regimen for patients with *BRAF* mutant tumors or patients who harbor RAS mutant tumors for which cytoreduction is indicated. The molecular sub-analysis of the TRIBE trial demonstrated FOLFOXIRI plus bevacizumab is a feasible treatment option irrespective of RAS or *BRAF* status [5]; however, exploratory sub-analyses of randomized trials according to RAS showed inconsistent results for efficacy of triplet plus bevacizumab regimen in RAS mutant tumors [2, 6]. Few studies prospectively evaluated the FOLFOXIRI plus bevacizumab regimen for RAS mutant mCRC.

In Japan, a phase II trial of FOLFOXIRI plus bevacizumab using the dosage of Gruppo Oncologico Nord Ovest (GONO)-FOLFOXIRI has been performed in previously untreated patients with mCRC. The safety analysis showed high incidences of neutropenia and febrile neutropenia: grade 3 or 4 neutropenia, 72.5%; grade 3 febrile neutropenia, 21.7% [7]. In a phase I trial of FOLFOXIRI in Japanese patients, the modified dosage was shown to be feasible without impairing the activity [8]. However, the recommended dosage of the triplet-regimen for Japanese patients remains controversial.

We therefore conducted the present phase II trial to evaluate not only the safety but also the effectiveness of modified-FOLFOXIRI plus bevacizumab as first-line therapy in Japanese patients with mCRC who harbor RAS mutant tumors (JACCRO CC-11; UMIN000015152). The JACCRO CC-11 trial used the modified-dosage of the triplet regimen to evaluate if the regimen is more feasible than GONO-FOLFOXIRI plus bevacizumab without decreasing effectiveness.

## RESULTS

### Study population

A total of 64 patients were enrolled in this trial at 28 Japanese hospitals between October 2014 and August 2016. The full analysis set consisted of 62 patients, and 63 patients were included in the safety population because 1 of the 64 patients did not meet the eligibility criteria but received the protocol treatment, and another patient did not receive any course of treatment (Supplementary Figure 1). The patients’ characteristics are summarized in Table 1. All 62 patients in the full analysis set were assessed for treatment effectiveness, after a median follow-up of 10.2 months as of the cut-off date of September 2017. The median age of the patients was 62.5 years (range, 36–75), and 27% had right-sided tumors. Fifty-seven (92%) of the 62 patients had an Eastern Cooperative Oncology Group (ECOG) performance status (PS) of 0, 48 (77%) had synchronous metastases, and 14 (23%) had metachronous disease.

**Table 1:** Patients’ characteristics (*N*=62)

Characteristic	*N*	%
Gender		
Male	34	55
Female	28	45
Age (years)		
Median (range)	62.5 (36-75)	
Performance Status		
ECOG 0	57	92
ECOG 1	5	8
Site of primary tumor		
Right	17	27
Cecum	6	10
Ascending	8	13
Transverse	3	5
Left	45	73
Colon	17	27
Rectum	28	45
Diagnosis		
Metachronous	14	23
Synchronous	48	77
Number of metastatic sites		
0, 1	23	37
≥2	39	63
Metastatic Sites		
Liver	48	77
Lung	27	44
Para-aortic lymph nodes	10	16
Peritoneum	11	18
Previous adjuvant chemotherapy		
Yes	2	3
No	60	97
Resection of primary tumor		
Yes	38	61
No	24	39
RAS status		
*KRAS* exon2 mt	50	80
*KRAS* exon3 mt	1	2
*KRAS* exon4 mt	5	8
*NRAS* exon2 mt	3	5
*NRAS* exon3 mt	3	5
*NRAS* exon4 mt	0	0

Abbreviations: ECOG, Eastern Cooperative Oncology Group; mt, mutation.

A total of 52 patients discontinued the protocol treatment, and 10 patients continued to receive the treatment. The median number of cycles administered per patient as induction treatment was 12 (range, 1 to 17). Two patients received more than the 12 planned cycles at the investigator’s discretion, resulting in a protocol violation. The second cycle of treatment had to be delayed because of toxicity (mainly neutropenia) in 66% of the patients. Treatment was delayed by 2 or more weeks in 30% of the patients. Twenty-four (39%) patients were given the second cycle with a reduced dose of irinotecan, oxaliplatin, or both according to criteria for dosage adjustment in the protocol. At the time of this analysis, the mean relative dose intensities of fluorouracil, irinotecan, and oxaliplatin were 97%, 81%, and 79%, respectively. Among the 52 patients who discontinued the treatment, the main causes of treatment discontinuation were disease progression in 31 (50%) patients, conversion surgery in 11 (18%) patients, and delayed recovery from adverse events in 5 (8%) patients (Supplementary Table 1).

### Effectiveness

The investigators’ assessment of effectiveness according to Response Evaluation Criteria in Solid Tumors (RECIST), version 1.1, was complete response in 3 (4.8%) patients and partial response in 44 (71%) patients for an objective response rate (ORR) of 75.8% (95% CI 65.1%–86.5%). The remaining 13 (21%) patients had stable disease, thus achieving a disease control rate of 96.8% (95% CI 92.4%-100%). All results for response were confirmed by central review, without any revisions. In addition, an exploratory analysis of response according to primary tumor location showed that the ORR was higher in patients with left-sided tumors than in patients with right-sided tumors (82.2% vs. 58.8%) (Table 2).

**Table 2:** Tumor response and survival outcomes in the full analysis set (*N*=62)

Outcome	All	Right-sided tumor (n=17)	Left-sided tumor (n=45)
Tumor response n (%) [95% CI]			
Complete response	3 (4.8) [0-10.2]	2 (11.8)	1 (2.2)
Partial response	44 (71.0) [59.7-82.3]	8 (47.1)	36 (80.0)
Stable disease	13 (21.0) [10.8-31.1]	6 (35.3)	7 (15.6)
Progressive disease	1 (1.6) [0-4.7]	1 (5.9)	0 (0)
Not assessable	1 (1.6) [0-4.7]	0 (0)	1 (2.2)
Objective response n (%)	47 (75.8)	10 (58.8)	37 (82.2)
95% CI	65.1-86.5	35.4-82.2	71.1-93.4
Disease control n (%)	60 (96.8)	16 (94.1)	44 (97.8)
95% CI	92.4-100	82.9-100	93.5-100
Early Tumor shrinkage (%)	45 (73.8)^*^	11 (64.7)	34 (77.3)
95% CI	62.7-84.8	42.0-87.4	64.9-89.7
Depth of response			
Median (%)	49.2^**^	40.2	49.6
Range (%)	-28.7-100	-28.7-100	4.5-100
Median PFS (months)	11.5	10.1	11.9
95% CI	9.5-14.0	5.9 -16.2	9.5 -14.0

PFS, progression-free survival.

^*^Early tumor shrinkage in 61 patients with available data on early response.

^**^ Depth of response in 61 patients with available data.

**Figure 1:**
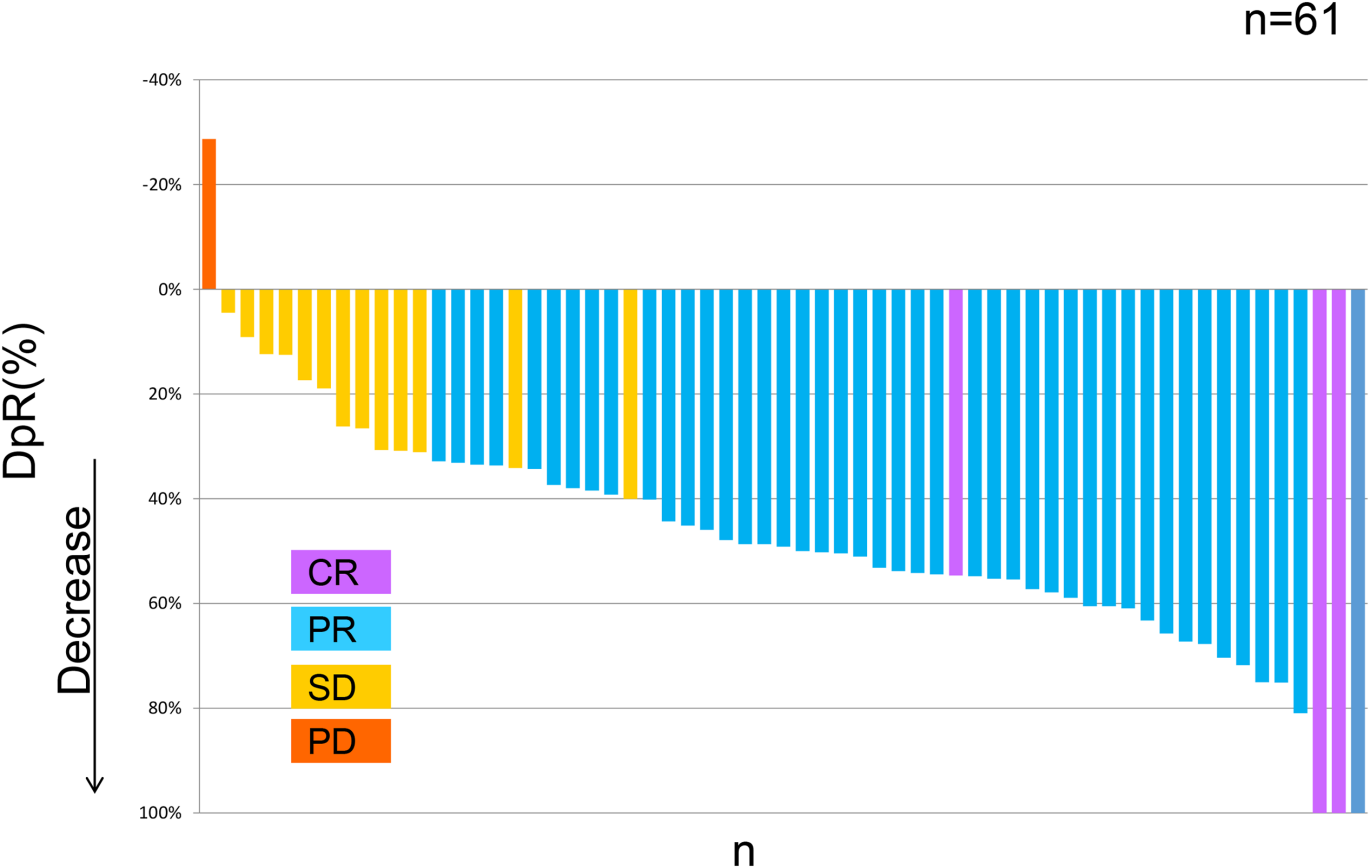
Waterfall plot of modified-FOLFOXIRI plus bevacizumab in 61 patients assessable for the depth of response

Among 61 patients in whom early tumor shrinkage (ETS) and depth of response (DpR) were assessable, ETS was obtained in 45 (73.8%) patients. Maximal tumor shrinkage occurred a median of 22 weeks (range 7 to 58) after starting treatment. The median DpR was 49.2% (range, -28.7% to 100%) (Figure 1). The progression-free survival (PFS) analysis was based on 37 events, and the median PFS was 11.5 months (95% CI, 9.5-14.0) (Table 2). Median overall survival (OS) was not reached because of the small number of events related to survival. Eleven (18%) of 62 patients could undergo surgical resection of metastatic sites.

### Safety

Safety was assessed according to the National Cancer Institute-Common Terminology Criteria for Adverse Events (NCI-CTC AE), version 4.0 in 63 patients belonging to the safety population, who had received at least one course of treatment, regardless of the eligibility criteria. The overall incidences of hematological and non-hematological toxicities are shown in Table 3. Grade 3 or 4 neutropenia was the most common adverse event, occurring in 34 (54%) of the 63 patients; moreover, 3 patients (5%) had grade 3 or 4 febrile neutropenia. Grade 3 or 4 non-hematological toxicities were hypertension (32%), diarrhea (13%), anorexia (11%), nausea (8%), and peripheral sensory neuropathy (2%). No treatment-related deaths occurred in the present study. A sub-analysis according to age indicated that elderly patients older than 70 years more frequently had grade 3 or 4 adverse events than did younger patients. No febrile neutropenia developed in patients 70 years of age or younger (Supplementary Table 2).

**Table 3:** Adverse events occurring in the safety population (*N*=63)

Adverse events	Grade 1 (%)	Grade 2 (%)	Grade 3 (%)	Grade 4 (%)	≥Grade 3 (%)
Leukopenia	11	35	24	5	29
Neutropenia	3	27	25	29	54
Anemia	24	21	6	0	6
Platelet count decreased	32	3	0	2	2
Nausea	44	22	8	0	8
Mucositis oral	38	11	3	0	3
Diarrhea	41	19	13	0	13
Fatigue	35	10	3	0	3
Paronychia	2	0	0		
AST increased	37	5	0	0	0
ALT increased	41	8	0	0	0
Hyponatremia	29	0	3	0	3
Hypokalemia	10	0	5	2	6
Proteinuria	32	14	3		3
Peripheral sensory neuropathy	54	35	2	0	2
Hypertension	13	33	32	0	32
Febrile neutropenia			2	3	5
Infusion-related reactions	0	2	0	0	0

We additionally performed an exploratory analysis of the irinotecan-related toxicity according to *UGT1A1* genotypes in the safety population. There was no statistically significant difference in the frequency of severe neutropenia among the 3 genotypes of *UGT1A1* (^*^1/^*^1, ^*^1/^*^28 or ^*^1/^*^6, and ^*^28/^*^28, ^*^6/^*^6, or ^*^28/^*^6), although the frequency was numerically higher in the ^*^28/^*^28, ^*^6/^*^6, or ^*^28/^*^6 genotype groups. The frequency of febrile neutropenia did not differ significantly among the genotypes (Supplementary Table 3).

## DISCUSSION

Our study demonstrated that modified-FOLFOLXIRI plus bevacizumab was an active first-line treatment in Japanese patients with mCRC who harbor RAS mutant tumors. The results in terms of the ORR met the primary endpoint: the response rate of 76% was comparable to the rates in previous phase II and III trials of GONO-FOLFOXIRI [1, 9]. In addition, the safety analysis indicated that the triplet regimen, including modified doses of each drug, was more feasible. To our knowledge, this is the first study to confirm the impact of first-line triplet therapy plus bevacizumab treatment in patients with RAS mutant mCRC.

Several international trials evaluating FOLFOXIRI plus bevacizumab in patients with mCRC have shown consistent results, supporting the effectiveness of the intensive chemotherapy. In the present phase II trial, the ORR of 75.8% was similar to the ORR obtained in the previous GONO-phase II trial (77%) [9], indicating that the intensive treatment is active in Asian patients. The subgroup analysis according to *BRAF* and RAS status in the TRIBE trial demonstrated that the benefits of FOLFOXIRI plus bevacizumab were consistent across all molecular subgroups [5]. However, in the phase II trial of triplet therapy plus bevacizumab, ORR and median PFS were numerically better in patients with RAS wild-type tumors than those with RAS mutant tumors [9]. In an exploratory sub-analysis of the CHARTA trial according to RAS status and tumor sidedness, median PFS was 14.0 months in patients with RAS mutant tumors as compared with 9.5 months in patients with RAS wild-type tumors among patients with right-sided primary tumors [6]. In a sub-analysis of the STEAM trial according to RAS status, the Kaplan-Meier curves of PFS appeared to be better in RAS wild-type than in RAS mutants among patients who received a sequential-FOLFOXIRI regimen, while there was no apparent difference in PFS between RAS wild-type tumors and mutant tumors among patients treated who received a concurrent-FOLFOXIRI regimen, suggesting that continuous exposure to the three drugs in the FOLFOXIRI regimen might be more beneficial in patients with RAS mutant mCRC [2]. The effectiveness of triplet therapy plus bevacizumab for RAS mutant tumors has been proven retrospectively by the sub-analyses of clinical trials. To our knowledge, ours is the first study to prospectively evaluate the effectiveness of FOLFOXIRI plus bevacizumab in patients with RAS mutant mCRC, thereby providing valuable evidence that triplet therapy plus bevacizumab is an attractive treatment for mCRC harboring RAS mutation.

In the TRIBE trial, FOLFOXIRI consisted of 165 mg/m^2^ irinotecan, 85 mg/m^2^ oxaliplatin, and 3200 mg/m^2^ continuous 5-fluorouracil in combination with bevacizumab (5 mg/kg) [1]. In the first pilot study, the GONO identified 175 mg/m^2^ irinotecan, 100 mg/m^2^ oxaliplatin, and 3800 mg/m^2^ 48-h chronomodulated continuous infusion 5-fluorouracil as the recommended dose of FOLFOXIRI [10]. Subsequently, however, the schedule was modified to the current version owing to the frequent occurrence of severe hematological toxicity in a phase II trial [11]. The incidences of grade 3 or 4 neutropenia, diarrhea, peripheral sensory neuropathy, and febrile neutropenia were respectively 50%, 19%, 5%, and 9% in the TRIBE trial [1]. In the Japanese phase I trial of FOLFOXIRI plus bevacizumab, GONO-FOLFOXIRI appeared to be tolerated by Japanese patients [12]; however, in a subsequent phase II trial, high incidences of neutropenia and febrile neutropenia were observed: 73% of patients had grade 3 or 4 neutropenia, and 22% had febrile neutropenia [7]. In particular, the incidence of grade 4 neutropenia was significantly higher in patients with UGT1A1^*^1/^*^6 genotype in the QUATTRO trial that evaluated GONO-triplet regimen plus bevacizumab for Japanese mCRC patients [13]. This might indicate that the dosage used in the TRIBE trial is not feasible for Japanese patients, specially with heterozygous of *UGT1A1* genotype. On the other hand, a phase I trial of FOLFOXIRI in Japanese patients with mCRC revealed that a modified-dosage (150 mg/m^2^ irinotecan, 85 mg/m^2^ oxaliplatin, and 2400 mg/m^2^ 5-fluorouracil) is more suitable for Japanese patients [8]. The JACCRO CC-11 trial evaluated the modified dosage of the triplet regimen and found it to be more feasible than the GONO-FOLFOXIRI regimen, without impacting efficacy. There was no difference in hematological toxicity between *UGT1A1* wild genotype and heterozygous patients in our study; therefore, the triplet regimen comprised of modified dosage of irinotecan may be more suitable for Japanese patients harboring UGT1A1^*^1/^*^6 or ^*^1/^*^28 genotype. Our results suggest that modified-FOLFOXIRI plus bevacizumab is a treatment option for Japanese patients with mCRC who receive triplet-based chemotherapy.

Our study also prospectively evaluated ETS and DpR in all of the subjects. ETS was confirmed in 74% of the 61 assessable patients in whom response was also evaluated by an external review board. The TRIBE trial reported an ETS of 63%, and the ETS ranged from 62% to 69% in the CRYSTAL, OPUS, and FIRE-3 trials [14, 15]. Sub-analyses of the TRIBE and FIRE-3 trials have revealed that patients who had an early response had better survival time than patients without ETS [15]; however, evaluation of ETS and DpR was not pre-planned in the TRIBE trial. The results of our phase II trial, which included an evaluation of the predictive value of ETS, may potentially show that treatment with FOLFOXIRI plus bevacizumab positively correlates with ETS or DpR and survival time.

Our trial had several limitations. The follow-up time was relatively short for evaluation of PFS and it was too short to analyze median OS, although the primary endpoint was met. In addition, the number of patients with events is considered insufficient for the evaluation of PFS and OS. However, the 64 patients enrolled in this study provided sufficient statistical power to draw final conclusions regarding effectiveness according to calculations based on the results of previous FOLFOXIRI-related studies [16]. The impacts of ETS and DpR on survival time should be evaluated after longer follow-up in future studies.

In conclusion, this prospective phase II trial demonstrated for the first time that FOLFOXIRI plus bevacizumab is an active first-line treatment for patients with mCRC who harbor RAS mutant tumors. In addition, our results suggest that modified-FOLFOXIRI plus bevacizumab might become an alternative regimen of triplet chemotherapy for mCRC in Japan.

## MATERIALS AND METHODS

### Patient population

The eligibility criteria of this trial were a histologically confirmed diagnosis of adenocarcinoma of the colon or rectum; a *KRAS* (exon 2, 3, or 4) or *NRAS* (exon 2, 3, or 4) mutant tumor with unresectable metastases; at least one measurable lesion of ≥10 mm or a residual nonmeasurable lesion according to the RECIST, version 1.1; adequate bone marrow function (hemoglobin concentration ≥9.0 g/dl, neutrophil count >1,500/mm^3^, platelet count >100,000/mm^3^), hepatic function, and renal function; an ECOG PS of 0–1 if patients were 70 years of age or younger, or 0 if they were 71 to 75 years of age; previous adjuvant chemotherapy had ended more than 12 months before the first relapse; no major surgical procedure within 28 days before treatment; no clinically significant cardiovascular disease; no evidence of proteinuria or coagulopathy; no thromboembolic or hemorrhagic events in the previous 6 months; and no current therapeutic treatment with anticoagulants. We excluded patients with any of the following conditions: uncontrolled infection, massive ascites, pleural effusion, symptomatic brain metastases, other malignancies within 5 years before enrollment (with the exception of early carcinoma that has been treated with curative intent), a history of palliative chemotherapy for metastatic disease, previous treatment with irinotecan or bevacizumab, or peripheral neuropathy of grade 1 or higher according to the NCI-CTCAE, version 4.0. The study was approved by the ethics committee at each participating center and was performed in accordance with the declaration of Helsinki. All patients provided written informed consent before enrollment. Fifty-three centers finally participated in this trial.

### Treatment

The treatment consisted of two phases: induction treatment and maintenance treatment. Induction treatment, given intravenously, consisted of bevacizumab 5 mg/kg on day 1 (the first infusion was delivered over the course of 90 minutes, the second infusion over the course of 1 hour, and subsequent infusions over the course of 30 minutes) plus modified-FOLFOXIRI (irinotecan 150 mg/m^2^ of body surface area [BSA] over the course of 1 hour on day 1, immediately followed by oxaliplatin 85 mg/m^2^ of BSA and l-leucovorin 200 mg/m^2^ of BSA infused concomitantly over the course of 2 hours, and then followed by a continuous 46-hour infusion of fluorouracil at a dose of 2400 mg/m^2^ of BSA on day 1). Induction treatment was administered every 2 weeks for a maximum of 12 cycles. Fewer cycles were given if there was evidence of disease progression, unacceptable toxicity, or withdrawal of consent. Maintenance treatment consisted of bevacizumab 5 mg/kg, l-leucovorin 200 mg/m^2^ of BSA, and fluorouracil 2400 mg/m^2^ of BSA, administered every 2 weeks until tumor progression, unacceptable toxicity, or patient refusal.

Treatment was continued until disease progression or unacceptable toxic effects occurred, a complete response was achieved, surgical resection became possible, or the patient requested or the physician decided that therapy should be withdrawn. Dose modification of chemotherapy was permitted according to the protocol-defined criteria. In patients with grade 3 or 4 allergic or hypersensitivity reactions, oxaliplatin was permanently discontinued.

### Assessments of efficacy and toxicity

The primary endpoint of the current study was the ORR (complete or partial response). Secondary endpoints were PFS, OS, safety, ETS, and DpR, defined as chronological tumor shrinkage (percent change in size of target lesions as compared with the baseline value) as evaluated every 8 weeks until disease progression. Response and progression were assessed on the basis of investigator-reported measurements, which were subsequently confirmed by central review according to RECIST, version 1.1. Computed tomography or magnetic resonance imaging had to be repeated every 8 weeks. The investigators at each center were required to provide the imaging files for a central revision of response or progression. The PFS was based on disease progression as determined by the board or on death from any cause, censoring data on patients who had not had progression at the date of cut-off or who discontinued the protocol treatment because of surgical resection or toxicity. The OS was calculated from the day of starting treatment until the day of death from any cause, censoring data on patients who had not died as of the last date they were known to be alive. Toxicity was assessed every cycle according to the NCI-CTC AE, version 4.0.

ETS was defined as a minimal tumor reduction of 20% at 8 weeks, while DpR was defined as the percentage of tumor shrinkage, based on the longitudinal diameters of target lesions according to RECIST, version 1.1 at the lowest point (nadir) as compared with the baseline values. A DpR of 100% indicates the complete disappearance of all target tumor lesions. If there is no change in tumor size, the DpR is zero; if tumor volume increases, the DpR is assigned a negative value [15].

### Statistical analysis

The response was evaluated among patients in the full analysis set who fulfilled all of the eligibility criteria and received at least one course of treatment. The 95% confidence intervals (CI) were calculated for the ORR and ETS using normal approximation to the binomial distribution. The Kaplan–Meier method was used to estimate PFS and OS. Distributions of time-to-event variables for both PFS and OS were estimated with the Greenwood’s formula. This phase II trial was designed to have a target activity level of 70% and a minimum activity level of 40%, with an α error of 0.05 and a β error of 0.10 on the basis of the results of the TRIBE and GONO studies, which reported a confirmed ORR of 65% to 77% for FOLFOXIRI plus bevacizumab [1, 9]. We estimated a minimum of 56 patients and planned to enroll a total of 60 patients so as to allow for a patient ineligibility rate of about 5%. Treatment was judged to be promising if the lower limit of the 95% CI exceeded 40%.

Statistical analyses were carried out using JMP 9.0.3 software (SAS Institute, Cary, NC, USA). This trial has been completed and is registered with UMIN, number 000015152.

## SUPPLEMENTARY MATERIALS FIGURE AND TABLES


