# Correction: A phase II trial of 1st-line modified-FOLFOXIRI plus bevacizumab treatment for metastatic colorectal cancer harboring RAS mutation: JACCRO CC-11

**DOI:** 10.18632/oncotarget.25775

**Published:** 2018-07-06

**Authors:** Hironaga Satake, Yu Sunakawa, Yuji Miyamoto, Masato Nakamura, Hiroshi Nakayama, Manabu Shiozawa, Akitaka Makiyama, Kazuma Kobayashi, Yutaro Kubota, Misuzu Mori, Masahito Kotaka, Akinori Takagane, Masahiro Gotoh, Masahiro Takeuchi, Masashi Fujii, Wataru Ichikawa, Takashi Sekikawa

**Affiliations:** ^1^ Department of Medical Oncology, Kobe City Medical Center General Hospital, Kobe, Hyogo, 650-0047, Japan; ^2^ Department of Clinical Oncology, St. Marianna University School of Medicine, Kawasaki, Kanagawa, 216-8511, Japan; ^3^ Department of Gastroenterological Surgery, Graduate School of Medical Sciences, Kumamoto University, Kumamoto, 860-8556, Japan; ^4^ Aizawa Comprehensive Cancer Center, Aizawa Hospital, Matsumoto, Nagano, 390-8510, Japan; ^5^ Department of Surgery, Nagoya Medical Center, Nagoya, Aichi, 460-0001, Japan; ^6^ Department of Gastrointestinal Surgery, Kanagawa Cancer Center, Yokohama, Kanagawa, 241-0815, Japan; ^7^ Department of Hematology/Oncology, Japan Community Healthcare Organization Kyushu Hospital, Kitakyusyu, Fukuoka, 806-8501, Japan; ^8^ Department of Surgery, Nagasaki University Graduate School of Biomedical Sciences, Nagasaki, 852-8501, Japan; ^9^ Division of Medical Oncology, Department of Medicine, Showa University School of Medicine, Shinagawa-Ku, Tokyo, 142-8666, Japan; ^10^ Division of Clinical Oncology, Jichi Medical University, Shimotsuke-Shi, Tochigi, 329-0498, Japan; ^11^ Gastrointestinal Cancer Center, Sano Hospital, Kobe, Hyogo, 655-0031, Japan; ^12^ Department of Surgery, Ha kodate Goryoukaku Hospital, Hakodate, Hokkaido, 040-8611, Japan; ^13^ Cancer Chemotherapy Center, Osaka Medical College Hospital, Takatsuki, Osaka, 569-8686, Japan; ^14^ Department of Clinical Medicine (Biostatistics), Kitasato University School of Pharmacy, Minato-ku, Tokyo, 108-8641, Japan; ^15^ Department of Digestive Surgery, Nihon University School of Medicine, Itabashi-ku, Tokyo, 173-8610, Japan; ^16^ Division of Medical Oncology, Showa University Fujigaoka Hospital, Yokohama, Kanagawa, 227-8501, Japan

**This article has been corrected:** The corrected author name is given below:

**Wataru Ichikawa**

Original article: Oncotarget. 2018; 9:18811-18820. https://doi.org/10.18632/oncotarget.24702

