# Supplementary material for: A phase II trial of 1st-line modified-FOLFOXIRI plus bevacizumab treatment for metastatic colorectal cancer harboring RAS mutation: JACCRO CC-11
Source: Oncotarget. 2018 Apr 10;9(27):18811–20. doi: 10.18632/oncotarget.24702 (PMC5922357; doi:10.18632/oncotarget.24702)
Supplement: Supplementary file 1 [file oncotarget-09-18811-s001.pdf]

A phase II trial of 1st-line modified-FOLFOXIRI plus bevacizumab treatment for metastatic colorectal cancer harboring RAS mutation: JACCRO CC-11

SUPPLEMENTARY MATERIALS

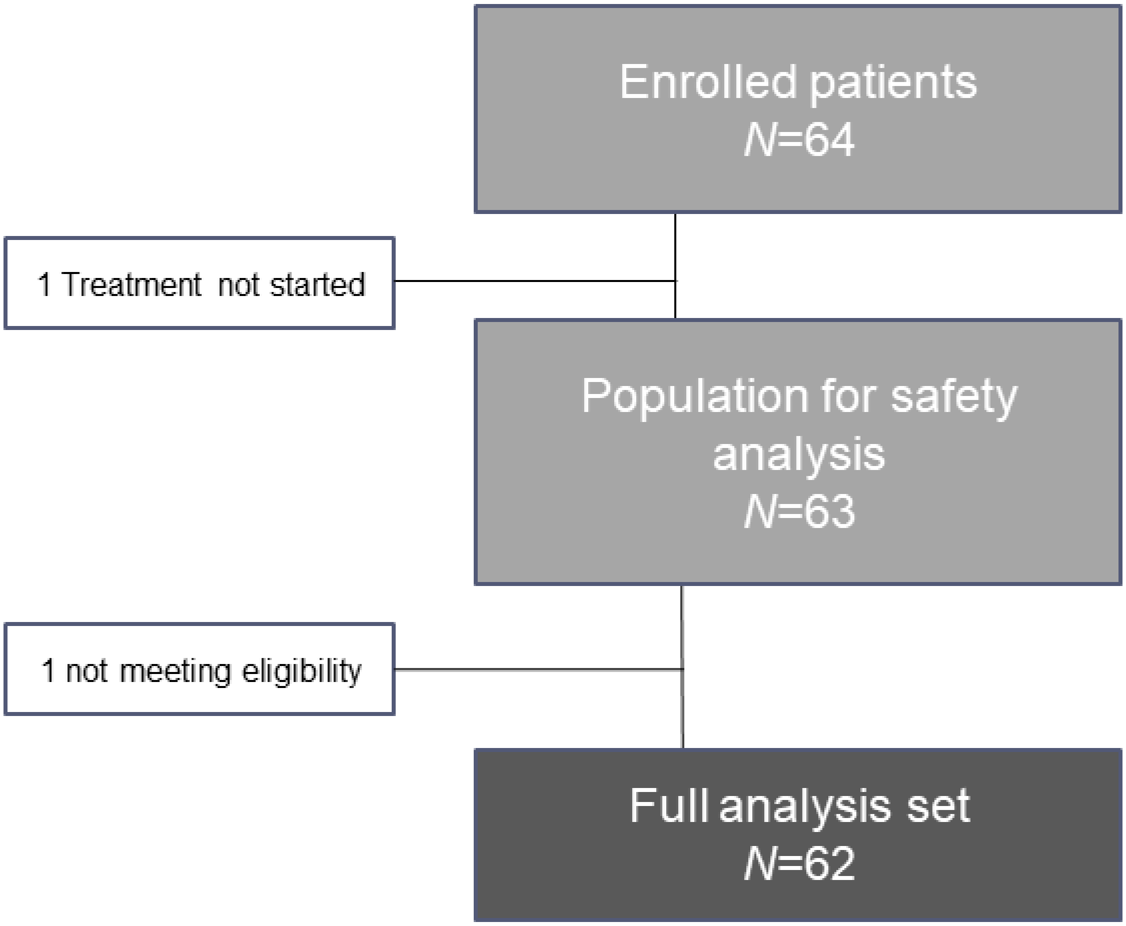

Supplementary Figure 1: Consort diagram of the study.

Supplementary Table 1: Reasons for protocol treatment discontinuation

	***N*=62**
Discontinuation of treatment	52
**Reasons**	
Disease progression	31
Convert to surgery	11
Delayed recovery from adverse events	5
Adverse event	2
Refusal by patient	1
Others	2
Treatment-related death	0

Supplementary Table 2: Adverse events according to age

Common adverse events	≤70 (n=55)		>70 (n=8)	
	All Grades (%)	≥Grade 3 (%)	All Grades (%)	≥Grade 3 (%)
Leukopenia	71	25	100	50
Neutropenia	82	51	100	75
Platelet count decreased	33	2	63	0
Anemia	49	7	63	0
Hypoalbuminemia	53	2	75	0
Hyponatremia	29	2	50	13
Hypokalemia	15	5	25	13
Proteinuria	44	2	88	13
Anorexia	78	9	88	25
Diarrhea	71	11	88	25
Febrile neutropenia	0	0	38	38
Hypertension	75	31	100	38

Supplementary Table 3: Adverse events according to *UGT1A1* status

Adverse event	*UGT1A1* status		
	*1/*1 (n=27)	*1/*28 or *1/*6 (n=18)	*28/*28, *6/*6, or *28/*6 (n=5)
Neutropenia			
Any Grade	21 (78%) (95%CI: 62.1 - 93.5)	15 (83%) (95%CI: 66.1 - 100)	5 (100%) (95%CI: 100 - 100)
p-value*		0.72	0.55
Grade 3-4	12 (44%) (95%CI: 25.7 - 63.2)	9 (50%) (95%CI: 26.9 - 73.1)	4 (80%) (95%CI: 44.9 - 100)
p-value*		0.77	0.33
Febrile Neutropenia			
Grade 3-4	0 (0%) (95%CI: 0 - 0)	2 (11%) (95%CI: 0 - 25.6)	0 (0%) (95%CI: 0 - 0)
p-value*		0.15	0
Diarrhea			
Any Grade	20 (74%) (95%CI: 57.5 - 90.6)	12 (67%) (95%CI: 44.9 - 88.4)	4 (80%) (95%CI: 44.9 - 100)
p-value*		0.74	1.00
Grade 3-4	5 (19%) (95%CI: 3.9 - 33.2)	2 (11%) (95%CI: 0 - 25.6)	2 (11%) (95%CI: 0 - 0)
p-value*		0.68	0.56

The 95% CI were calculated using normal approximation to the binomial distribution.
*Fisher's exact test (compared to *1/*1).
